# Effect of Sputtering Temperature on Fluorocarbon Films: Surface Nanostructure and Fluorine/Carbon Ratio

**DOI:** 10.3390/nano9060848

**Published:** 2019-06-03

**Authors:** Qi Zhao, Feipeng Wang, Kaizheng Wang, Guibai Xie, Wanzhao Cui, Jian Li

**Affiliations:** 1 State Key Laboratory of Power Transmission Equipment & System Security and New Technology, Chongqing University, Chongqing 400044, China; zhaoqi@cqu.edu.cn (Q.Z.); 20131002018@cqu.edu.cn (K.W.); lijian@cqu.edu.cn (J.L.); 2 National Key Laboratory of Science and Technology on Space Microwave, China Academy of Space Technology (Xi’an), Xi’an 710100, China; xieguibai@163.com (G.X.); cuiwanzhao@126.com (W.C.)

**Keywords:** fluorocarbon, surface nanostructure, sputtering temperature, surface properties

## Abstract

In this work, fluorocarbon film was deposited on silicon (P/100) substrate using polytetrafluoroethylene (PTFE) as target material at elevated sputtering temperature. Field emission scanning electron microscopy (FESEM), atomic force microscopy (AFM), Raman spectroscopy and X-ray photoelectron spectroscopy (XPS) were employed to investigate the surface morphology as well as structural and chemical compositions of the deposited film. The surface energy, as well as the polar and dispersion components, were determined by water contact angle (WCA) measurement. The experimental results indicated that increasing sputtering temperature effectively led to higher deposition rate, surface roughness and WCA of the film. It was found that the elevated temperature contributed to increasing saturated components (e.g., C–F_2_ and C–F_3_) and decreasing unsaturated components (e.g., C–C and C–CF), thus enhancing the fluorine-to-carbon (F/C) ratio. The results are expected aid in tailoring the design of fluorocarbon films for physicochemical properties.

## 1. Introduction

Fluorocarbon film, due to its superior hydrophobicity, low dielectric constant and small friction coefficient, is a promising material in various fields (e.g., self-cleaning coatings for perovskite solar cells, interlayer dielectrics in integrated circuits and lubricant coatings in micro-machines) [1,2,3]. Based on these attractive properties, a variety of approaches have been applied to fabricate fluorocarbon films, such as dielectric barrier discharge (DBD), ion beam sputter depositing (IBSD) and plasma-enhanced chemical vapor deposition (PECVD) [4,5,6]. However, drawbacks like induced impurities, small-scale deposition, as well as harmful and expensive raw materials (e.g., CF_4_, C_4_F_8_ and C_5_F_8_) restrict their extensive applications. Radio-frequency (RF) sputtering is quoted as an excellent alternative for fluorocarbon film fabrication owing to its capability of large-scale deposition, safe preparation process and excellent process controllability [7,8,9]. The sputtered fluorocarbon film exhibits improved physicochemical properties (e.g., lubrication, chemical inertness and thermal stability) by controlling the fabrication process to form a structure similar to that of polytetrafluoroethylene (PTFE). The PTFE-like fluorocarbon film with improved physicochemical properties is expected and pursued in various fields [10,11].

The deposition of fluorocarbon films is controlled by various factors, including sputtering type [12,13,14], target material [15,16] and substrate temperature [17,18]—all of which govern the modulus, hardness and wettability by adjusting the elemental composition and surface morphology. Since the deposition is a plasma-associated process, much is known of the sputtering power [19], target–substrate distance [20,21] and gas conditions [22,23,24,25] that vary the plasma conditions, leading to various fluorocarbon film characteristics (e.g., electrical conductivity, scratch resistance and thermal stability). Additionally, the chamber temperature has been documented to affect the surface topography and wettability of fluorocarbon films [26]. The phenomenon of an elevated chamber temperature leading to plasma color transformation from light red to dark red is also observed in our experiments. As is well-known, the color change manifests variations of plasma species, density and temperature, etc. [27]. This prompted us to investigate the influence of sputtering temperature, which includes the chamber and substrate temperatures, on fluorocarbon film deposition. This work aimed to obtain full-scale understanding of the kinetic energies of argon ions, the collisions between argon atoms and ejected fragments (e.g., C–C, C–CF, C–F), as well as the adsorption capabilities of ejected fragments by discovering the variations of surface topography and elemental composition with sputtering temperature.

## 2. Materials and Methods

**Figure 1:**
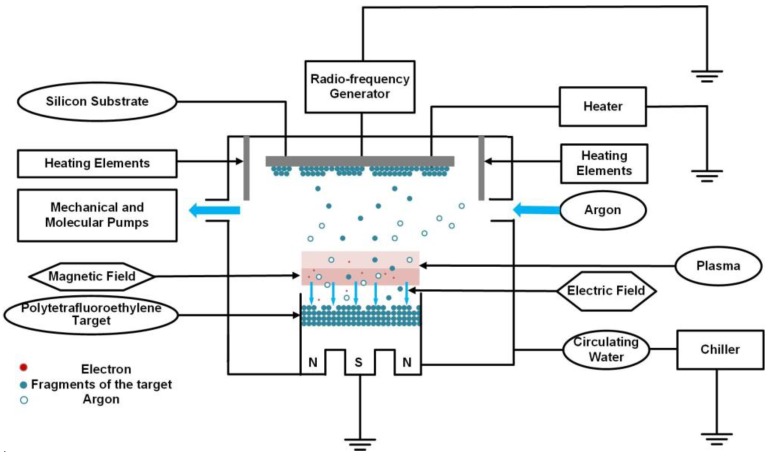
Schematic diagram of the magnetron sputtering system.

Figure 1 shows the RF (13.56 MHz) magnetron sputtering system for fluorocarbon film fabrication. The vacuum chamber of the sputtering system was in a “sputter-up” configuration with a PTFE target (purity 99.99%, diameter 3 inches, thickness 4 mm). The silicon substrate (size 4 × 4 mm^2^, thickness 0.5 mm) was mounted on top of the chamber with the capability of adjusting temperature (50–200 °C) by an installed heater and a thermocouple. The stainless-steel heating elements were fixed around the substrate with another thermocouple monitoring the chamber temperature.

The silicon substrate was ultrasonically cleaned by anhydrous alcohol and deionized water successively, and then dried in nitrogen before being loaded into the chamber. The chamber was evacuated to a base pressure of 5 × 10^−4^ Pa. The PTFE target was pre-sputtered in argon plasma for 15 min in order to remove excessive oxide surface layer. The sputtering was conducted in argon plasma at a target–substrate distance of 100 mm, a working pressure of 0.5 Pa and a discharge power of 80 W for 1 h. Both the chamber and substrate temperatures were controlled at the same value. Each experiment was done at a fixed temperature of 50, 100, 150 and 200 °C.

The film thickness was an average value of five measurements taken by a surface profiler (Dektak XT, Bruker, Germany). Field-emission scanning electron microscopy (FESEM, JSM-7800F, JEOL, Tokyo, Japan) with a beam accelerating voltage of 20 kV was applied to inspect the surface morphology of fluorocarbon films. An atomic force microscope (AFM, MFP-3D-BIO, Asylum Research, Goleta, CA, USA) working in contact mode was utilized to measure the arithmetic average of the roughness profile (R_a_). The AFM provided quantitative information about surface structures through a mechanical tip which scans and senses the surface. The static water contact angle (WCA) was examined on a WCA goniometer (SDC-100, SINDIN, Chengdu, China). Both contact angles of water and methylene iodide with the droplet volume of 10 μL were measured at room temperature. The polar component (γ_s_^p^) and dispersion component (γ_s_^d^) of the surface free energy (γ_s_^owk^) were calculated by applying the contact angles of water and methylene iodide to the Owens–Wendt–Kaelble (OWK) approach and Young’s equation (Equations (1) and (2)) [28].

γ_s_^owk^ = γ_s_^p^+γ_s_^d^ (1)

γ_L_·(1 + cosθ) = 2[(γ_s_^d^γ_L_^d^)^1/2^ + [(γ_s_^p^γ_L_^p^)^1/2^] (2)

In Equation (2), the parameters of θ and γ_L_ indicate the measured contact angles and the known surface free energy of water and methylene iodide, respectively. The structural changes of fluorocarbon films were determined by Raman spectroscopy (LabRAM HR Evolution, HORIBA Jobin Yvon S.A.S, Palaiseau, France) with laser wavelength of 325 nm. Based on the interaction of light with chemical bonds within the films, the non-destructive light scattering technique provides detailed spectra, which demonstrates the intensity and wavelength position of the Raman scattered light. The chemical composition was evaluated through X-ray photoelectron spectroscopy (XPS, ESCALAB 250Xi, Thermo Scientific, Waltham, MA, USA) with Al K_α_ radiation (1486.6 eV) at a power of 25 W. Charging of the films that resulted from photoemission was calibrated using adventitious carbon referencing (C 1s, 284.6 eV). The spot size of the X-ray beam was 500 × 500 μm^2^ in each case.

## 3. Results and Discussion

**Figure 2:**
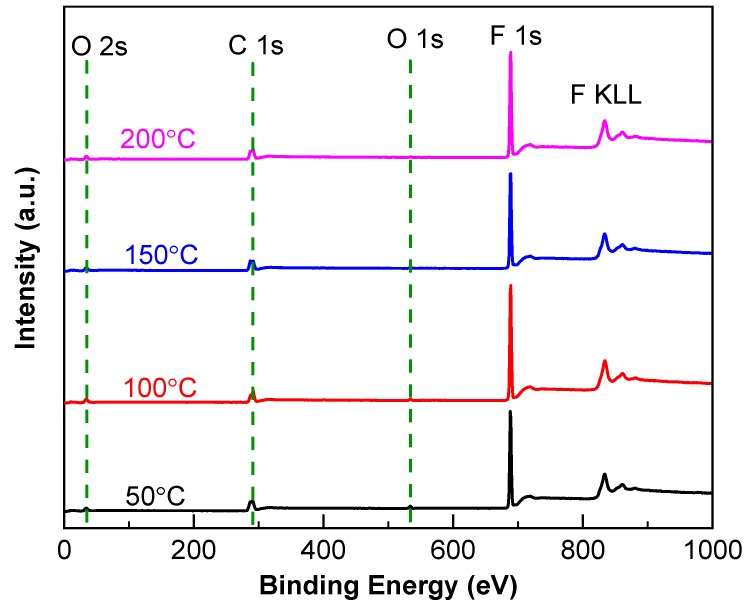
Full-scan XPS spectra of fluorocarbon films as a function of sputtering temperature.

Figure 2 exhibits the overall XPS spectra of fluorocarbon films with elevated temperature. The dominant peak of F 1s with small peaks of C 1s, O 1s and O 2s in XPS spectra verify the successful deposition of the films on the substrate. Compared with the molecular formula of PTFE ([C_2_F_4_]_n_), the appearance of weak O 1s and O 2s peaks indicates that a small amount of oxygen was present in fluorocarbon films, which may be due to the residual contaminations on the substrate, as well as the oxidation of the films when exposed to the atmosphere. Furthermore, the peak intensities of C 1s and F 1s increased while those of O 1s and O 2s decreased with the elevated temperature. The phenomenon was probably caused by the removal of adsorbed contaminants from the substrate, which resulted from the increased substrate temperature. Accordingly, the elevated temperature was beneficial for improving the purity of the fluorocarbon films.

**Figure 3:**
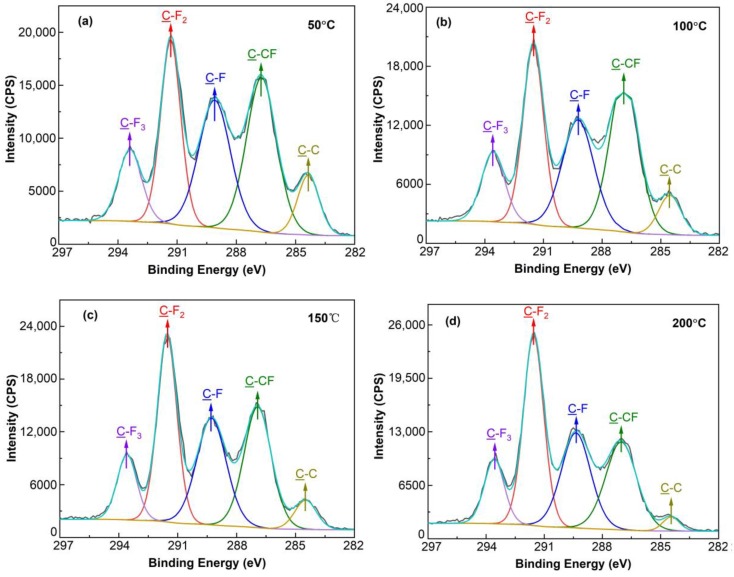
The C 1s spectra of fluorocarbon films deposited at the sputtering temperature of 50 °C (**a**), 100 °C (**b**), 150 °C (**c**) and 200 °C (**d**).

The XPS spectra related to the C 1s signal (Figure 3) were divided into five peaks at the binding energies of 284.2, 286.8, 289.7, 291.5 and 293.5 eV, suggesting the existence of C–C, C–CF, C–F, C–F_2_ and C–F_3_ bonds, respectively [14,29,30,31,32]. The expression of C–F, C–F_2_ and C–F_3_ means that the carbon atoms are linked to one, two and three fluorine atoms, respectively, while C–CF indicates the connection of one carbon atom to another carbon atom, which is bound with a fluorine atom. In addition, C–C demonstrates that only two carbon atoms are linked together. The area under each peak indicates the relative presence of each bond type.

**Figure 4:**
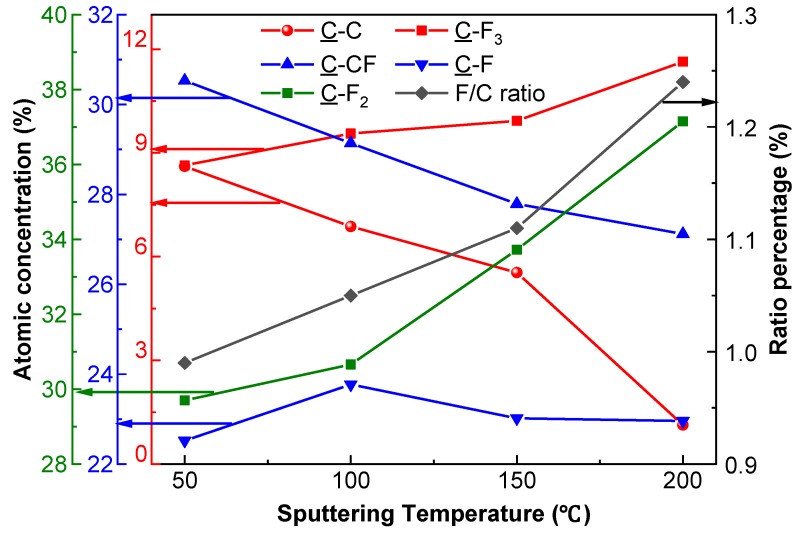
Atomic concentrations of chemical bonds and F/C ratio of fluorocarbon films.

The elevated temperature was beneficial for the enhanced atomic concentrations of C–F_2_ and C–F_3_, which increased to 37.15% and 11.64%, respectively (Figure 4). Meanwhile, the atomic concentrations of C–C and C–CF dramatically reduced down to 1.13% and 27.12%, respectively. However, the atomic concentration of C–F fluctuated, first increasing from 22.52% to 23.77%, and then decreasing from 23.77% to 22.96%, via 23.02%. The calculated F/C ratio of fluorocarbon films exhibited an increasing trend, from 0.99 to 1.24 (via 1.05 and 1.11) with the elevation of temperature from 50 to 200 °C, via 100 and 150 °C.

For the PTFE target characterized by long chains of fluorinated carbons, [CF_2_-CF_2_]_n_ served as the bulk of the chain. The appeared chemical bonds of C–C, C–CF and C–F_x_ (x = 1, 2, 3) indicate that the chain had broken due to the collisions between the target and argon. As the bond dissociation energy of C–C (618.3 ± 15.4 kJ/mol) is higher than that of C–F (513.8 ± 10.0 kJ/mol), the formation of C–C bonds is much easier than the formation of C–F bonds [33]. The survival competition between the saturated components (SCs, e.g., C–F_3_, C–F_2_) and the unsaturated components (UCs, e.g., C–C, C–CF) on the substrate indicates that the formation of SCs requires a higher momentum transfer. Therefore, the proportion of SCs increased with the elevated substrate temperature. The survival competition between UCs and SCs was confirmed by the increased proportions of C–F_2_ and C–F_3_, as well as the relatively decreased proportions of C–C and C–CF. As the structure of the PTFE target is long continuous chains with fluorinated carbon twisted into a helix, the fluorine sheath of PTFE exhibits a compact “capsule” structure. The fluorine sheath consists of C–F bonds. The initial breaking of C–F bonds offers opportunities for argon ions to collide with C–C bonds inside the sheath, resulting in the breaking of C–C bonds. Therefore, although the elevated substrate temperature was beneficial for the formation of a higher proportion of C–F bonds on the substrate, the atomic concentration of C–F fluctuated with the sputtering temperature. The increased F/C ratio was consequently caused by the formation of a higher proportion of SCs and a lower proportion of UCs on the substrate. Accordingly, the elevated sputtering temperature contributed to increasing the F/C ratio, which favors the formation of PTFE-like fluorocarbon films with superior physicochemical properties [34,35].

**Figure 5:**
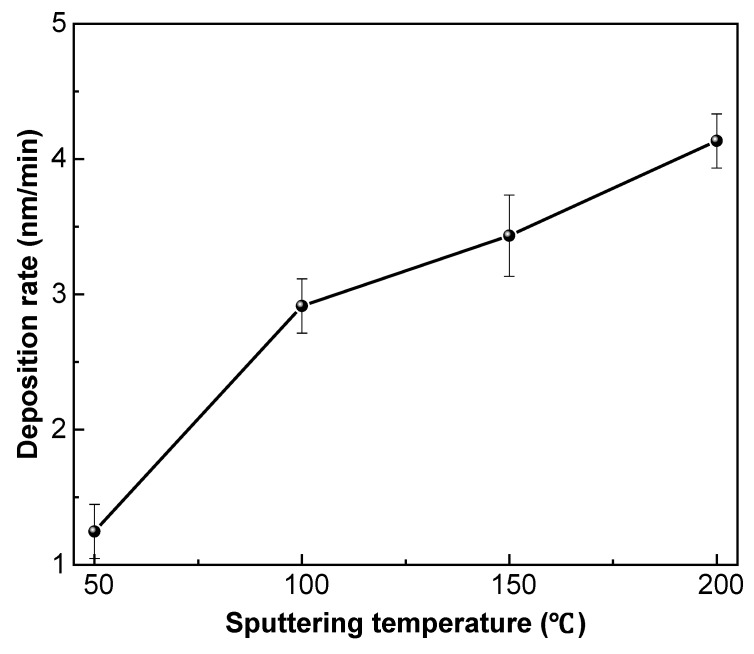
Deposition rate of fluorocarbon films as a function of sputtering temperature (error bars indicate 95% confidence intervals).

Figure 5 shows a nonlinear relationship between sputtering temperature and the deposition rate of fluorocarbon films. The elevated temperature from 50 to 200 °C effectively increased the deposition rate from 1.25 to 4.13 nm/min, via 2.91 and 3.43 nm/min. The sputtering temperature covered both the chamber and substrate temperatures. The elevated chamber temperature induced the increased temperature of argon ions. Higher temperature of argon ions prolongs its mean free path (λ′_fp_), according to Equations (3) and (4). In these equations, N relates to the density of argon ions. P_g_ and T_g_ refer to the pressure and temperature of argon ions. K_B_ is the Boltzmann constant. σ indicates the geometric cross section between argon ions and other particles (e.g., argon atoms and ejected fragments) during collisions.

N = P_g_/(K_B_·T_g_) (3)

λ = 1/(N·σ)(4)

E_i_ = 2(λ′_fp_/L)·(e·V_c_) (5)

Based on Equation (5), the prolonged λ′_fp_ increases the average energy (E′_i_) of argon ions. L is the distance between the cathode and the anode of the RF sputtering system. V_c_ indicates the cathode fall voltage and e refers to the electron charge [36]. The elevated chamber temperature should be beneficial for increasing the ion temperature, as well as the ion energy. However, the ion energy gained from the higher chamber temperature is less than 1% of that from the power supply. This phenomenon guides us to focus the process of the fragments from target to substrate, which could be strongly influenced by the elevated chamber temperature.

The higher chamber temperature increases the temperature of ejected fragments, resulting in their prolonged mean free path (λ″_fp_). The prolonged λ″_fp_ decreases the number of effective collisions between ejected fragments and other particles (e.g., argon ions and atoms), which is helpful to reduce the kinetic energy loss of ejected fragments. Furthermore, the prolonged λ″_fp_ is beneficial for the increased average energy (E″_i_) of ejected fragments, thus contributing to the successful deposition of ejected fragments on the substrate [37].

Elevated substrate temperature has been reported to have a negative effect on fluorocarbon film deposition, due to the negative apparent activation energy, which promotes desorption rather than adsorption of ejected fragments on the substrate [10]. The promoted desorption reduces the sticking coefficient of ejected fragments, thus weakening their adhering capabilities to the substrate [17]. The sticking coefficient depicts a probability for the ejected fragments to be trapped on the surface of the silicon substrate through losing their kinetic energy by transferring their energy to the silicon atoms. However, the reduced sticking coefficient tends to be offset by the increased E″_i_ [29]. In addition, the atomic concentrations of larger fragments (e.g., C–F_2_, C–F_3_) increases with the elevated sputtering temperature (Figure 4). As larger fragments exhibit a higher sticking coefficient than smaller fragments (e.g., C–C and C–CF), the increased atomic concentrations of larger fragments help to increase the sticking coefficient of fluorocarbon films [29]. Moreover, the elevated substrate temperature is beneficial for fluorocarbon film densification by enhancing the re-condensation process of ejected fragments on the substrate [38]. Accordingly, the elevated sputtering temperature is beneficial for more efficient fluorocarbon film deposition, through the decreased energy loss, improved sticking coefficient and enhanced re-condensation of ejected fragments.

**Figure 6:**
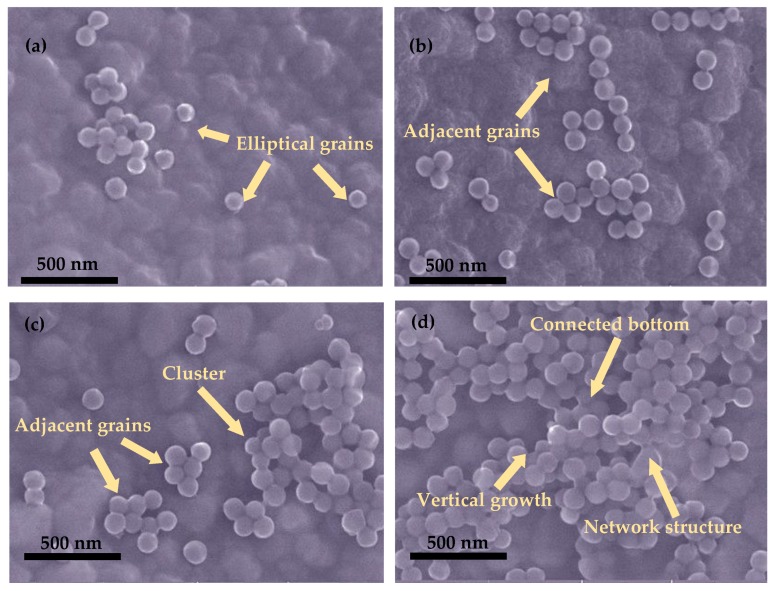
Surface morphology of fluorocarbon films deposited at various sputtering temperatures: (**a**) 50 °C, (**b**) 100 °C, (**c**) 150 °C, (**d**) 200 °C.

Figure 6 shows the nanostructural evolution of fluorocarbon films as a function of elevated sputtering temperature. It can be seen that the films consisted of almost spherical grains with an equivalent diameter of 100 nm. For the temperatures of 50 and 100 °C, the amount of grains was relatively small, which is consistent with the low deposition rate demonstrated in Figure 5. The grains were sparsely distributed on the substrate, with a tendency of adjoining each other at elevated temperatures. When the temperature increased to 150 °C, the adjacent grains pressed together and formed large numbers of clusters. At 200 °C, the bottoms of the clusters connected with each other and the top of that grew vertically, leading to the network structure formation of granular fluorocarbon films.

Based on the minimum surface area criterion, grains exhibit a spherical shape, with the lowest surface energy. The similar sizes of spherical grains indicate their formation in vapor phase. The surface morphology of granular films is primarily controlled by the mobility and diffusion of ejected fragments on the substrate. Owing to the increased E″_i_, the ejected fragments exhibited activated mobility, which contributed to the formation of adjacent grains. The improved diffusion of ejected fragments was achieved by the elevated substrate temperature, which favors grain accumulation and cluster densification [39]. Moreover, as the elevated sputtering temperature is beneficial for higher deposition rates, more grains formed on the substrate. The increased amount of grains reduces the average distance between them and contributes to their combination.

**Figure 7:**
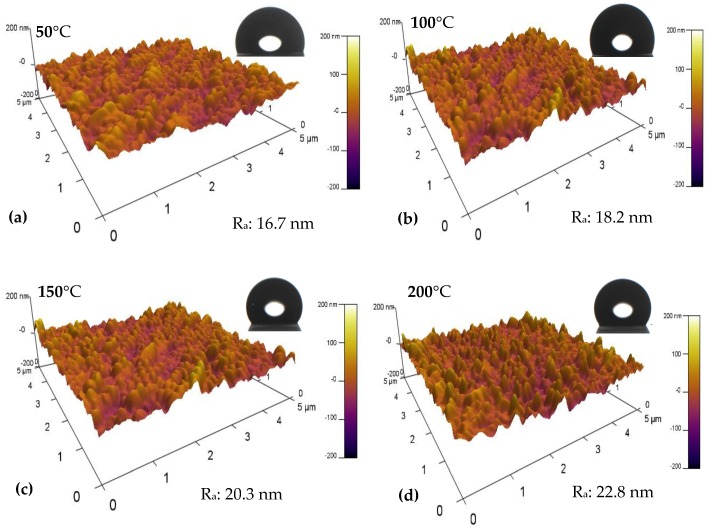
Atomic force microscopy (AFM) images and water contact angles (WCAs) of fluorocarbon films sputtered at 50 °C (**a**), 100 °C (**b**), 150 °C (**c**) and 200 °C (**d**).

The AFM images in Figure 7 indicate an increased R_a_ from 16.7 to 22.8 nm, via 18.2 and 20.3 nm with the elevated sputtering temperature. The insert images are corresponding WCAs of fluorocarbon films. Based on the measured contact angles, the γ_s_^OWK^, γ_S_^d^ and γ_S_^p^ were calculated and are listed in Table 1. As WCA of the reference silicon substrate is 68.2°, the introduction of fluorocarbon films on the substrate at 50 °C dramatically increased the WCA to 107.2°. The further elevated temperature contributed to a higher WCA from 112.1° to 125.1°, via 122.5°. Based on Equations (1) and (2), the calculated γ_s_^OWK^ decreased from 11.82 to 9.52 mJ/m^2^. Meanwhile, γ_S_^d^ and γ_S_^p^ dropped from 15.13 and 1.09 mJ/m^2^ down to 9.49 and 0.02 mJ/m^2^.

The elevated sputtering temperature was confirmed to promote the growth, motion and coalescence of fluorocarbon films according to their nanostructural evolution (Figure 6). For the sputtering temperature of 50 °C, clusters seldom formed on the substrate. Surface roughness mainly depends on the small amount of initially dispersed grains. With the increase of sputtering temperature from 100 to 150 °C, the grains gradually joined with each other, leading to the formation of clusters. As the size of clusters is larger than that of grains, the surface roughness was further enhanced. For sample 4, prepared at 200 °C, the granular fluorocarbon film showed a nanoscale network structure consisting of larger clusters. The formation of the network structure further enlarged the film’s surface roughness. Accordingly, the film surface roughness is determined by three factors (i.e., grain, cluster and network structure), which are dependent on the sputtering temperature.

The surface roughness and chemical composition of fluorocarbon films are considered to have a critical influence on the WCA [40]. According to the Cassie model, the WCA of a rough surface is a composite contact angle between water and the compound surface made of fluorocarbon film and air [41]. The elevated temperature from 50 to 200 °C increased the surface roughness through the deposition of grains and clusters, followed by the formation of the network structure. Compared with grains, clusters are more capable of trapping air. Furthermore, the network structure has the most powerful ability to trap air, thus preventing penetration of the droplet into the film surface. Accordingly, the elevated temperature contributed to enhanced surface roughness, leading to the increased WCA of fluorocarbon films.

Compared with the WCA of the silicon substrate, the increased WCA of fluorocarbon films was mainly attributed to the presence of fluorinated groups, which help to lower γ_s_^OWK^ due to their chemical inertness [42]. In addition, XPS results confirmed the increased fluorine-to-carbon (F/C) ratio, resulting in further reduced γ_s_^OWK^ of fluorocarbon films with elevated temperature (Figure 4). Therefore, both the enhanced surface roughness and the reduced γ_s_^OWK^ were responsible for the increased WCA of the films. Table 1 shows that the reduced γ_s_^OWK^ was mainly due to the decreased γ_S_^d^ of the fluorocarbon films. Overall, the γ_s_^OWK^ originates from the unbalanced forces between atoms or molecules inside and interface. The γ_S_^p^ is determined by different intermolecular forces (e.g., permanent, induced dipoles and hydrogen bonding), while the γ_S_^d^ known as London forces is caused by instantaneous dipole–induced dipole interactions. Specifically, the γ_S_^p^ reduced due to fewer unsaturated components (e.g., C–C and C–CF) in fluorocarbon films with the elevated temperature [43]. Compared with the slightly changed amount of C–F, the decreased amount of C–CF and increased amount of C–F_2_ contributed to reduce the γ_S_^d^ (Figure 4) [43,44].

**Figure 8:**
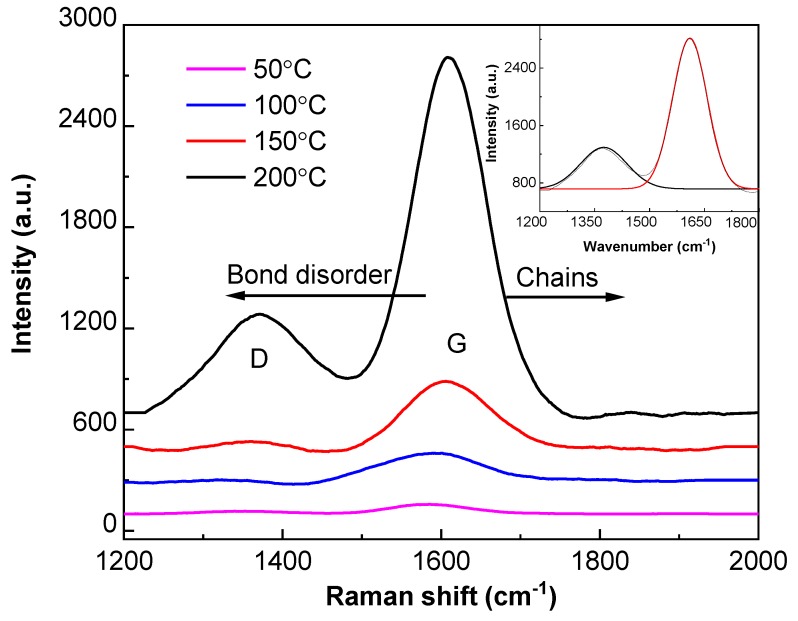
Raman spectra of fluorocarbon films deposited at different sputtering temperatures.

Figure 8 reports the Raman spectra (1200–2000 cm^−1^) detecting a G (graphite) peak and a D (disorder) peak. The G peak was broader and more asymmetric compared to the D peak. The peak intensities and positions were obtained by deconvolving the spectra. The deconvolved spectra revealed the intensity ratio of D and G peaks (I(D)/I(G)), which decreased from 0.74 to 0.46, via 0.66 and 0.60. Meanwhile, the corresponding G peak position shifted upward from 1585 to 1609 cm^−1^, via 1592 and 1606 cm^−1^, with the elevated temperature from 50 to 200 °C, via 100 and 150 °C.

The G peak in Figure 8 was attributed to the symmetric E_2g_ vibrational mode in graphite-like materials, while the D peak stemmed from the limitation in the graphite domain size induced by grain boundaries or imperfections, such as sp^3^ carbon and other defects [45]. The Raman spectra reflected the amorphous structure of fluorocarbon films with sputtering temperature ranging from 50 to 200 °C. The decreased intensity ratio of I(D)/I(G) and upshifting of the G peak position towards a higher wavenumber were attributed to the increased proportion of sp^3^ carbon atoms, accompanying more covalently bonded fluorine to carbon [46,47]. From the molecule kinematics viewpoint, the elevated sputtering temperature contributed to prolonging the mean free path of argon atoms and electrons, thus decreasing their collisions. The decreased collisions between argon atoms and electrons reduced the ionization rate of argon ions [48]. As the PTFE target consists of sp^3^ carbon atoms, the reduced ionization rate, together with the enhanced deposition rate of fluorocarbon films, promoted the formation of a higher proportion of sp^3^ carbon atoms (Figure 5) [49]. The increased proportion of sp^3^ carbon atoms is beneficial for the sp^2^ configuration of olefinic groups with high vibrational frequencies, which leads to the upshifting of the G peak position [46].

## 4. Conclusions

In this work, fluorocarbon film was deposited on silicon substrate at elevated sputtering temperatures from 50 to 200 °C, via 100 and 150 °C. The deposition rate of the films increased from 1.25 to 4.13 nm/min, and the surface roughness was enhanced from 16.7 to 22.8 nm. Based on the increased WCA of fluorocarbon films from 107.2° to 125.1°, the calculated γ_s_^OWK^ decreased from 16.22 to 9.52 mJ/m^2^, accompanied by both the reduction of γ_S_^d^ and γ_S_^p^. Besides the increased surface roughness, the enhanced F/C ratio was also responsible for the WCA increment. The elevated temperature enhanced the F/C ratio from 0.99 to 1.24, due to the increased proportions of saturated components and decreased proportions of unsaturated components. From the molecule kinematics viewpoint, the elevated temperature contributes to prolonging the mean free path of argon ions, argon atoms and ejected fragments, leading to reduced collisions inside the vacuum chamber and the improved sticking coefficient on the substrate. Therefore, elevated temperature contributes to promote fragment deposition on the substrate with increased F/C ratio.

## Figures and Tables

**Table 1:** Surface properties of fluorocarbon films.

Sputtering Temperature (°C)	R_a_ (nm)	WCA (°)	γ_s_^OWK^ (mN/m)	γ_s_^d^ (mN/m)	γ_s_^p^ (mN/m)
50	16.7	107.2	16.22	15.13	1.09
100	18.2	112.1	11.82	10.74	1.08
150	20.3	122.5	10.54	10.48	0.06
200	22.8	125.1	9.52	9.49	0.02
